# Understanding the links between cardiovascular and psychiatric conditions

**DOI:** 10.7554/eLife.84524

**Published:** 2022-12-02

**Authors:** Sonali Amarasekera, Prabhat Jha

**Affiliations:** 1 Dalla Lana School of Public Health, Epidemiology Division, University of Toronto Toronto Canada; 2 Centre for Global Health, Dalla Lana School of Public Health, University of Toronto Toronto Canada

**Keywords:** cardiovascular disease, psychiatric disorder, cohort, sibling, family design, Human

## Abstract

Individuals recently diagnosed with a cardiovascular disease are at higher risk of developing a mental illness, with mortality increasing when both conditions are present.

**Related research article** Shen Q, Song H, Aspelund T, Yu J, Lu D, Jakobsdóttir J, Bergstedt J, Yi L, Sullivan P, Sjölander A, Ye W, Fall K, Fang F, Valdimarsdóttir U. 2022. Cardiovascular disease and subsequent risk of psychiatric disorders: a nationwide sibling-controlled study. *eLife* **11**:e80143. doi: 10.7554/eLife.80143.

Cardiovascular diseases are the leading cause of mortality worldwide, accounting for approximately 32% of all deaths globally. Mental illnesses are similarly common, with approximately one in every eight individuals living with a mental health disorder in 2019 (World Health Organization, 2022). Given their high prevalence, these conditions are likely to exist alongside each other and this co-occurrence warrants rigorous scientific investigation.

The relationship between heart disease and mental illness is complex and bidirectional. For example, being diagnosed with heart failure can understandably cause stress and despair, and consequently elevate an individual’s risk of developing a major depressive disorder (Hare et al., 2014). Conversely, depressive disorders are known to manifest as sleep disturbances, reduced levels of physical activity and difficulty following health recommendations — all factors linked to an increased likelihood of developing cardiovascular conditions.

Evidence exists that the risks for mental and cardiovascular diseases increase in tandem (Schöttke and Giabbiconi, 2015; Ziegelstein, 2001). However, this body of work has important limitations that hinder drawing meaningful conclusions. For example, some studies only capture patient information at a single point in time, making it difficult to establish whether it was the cardiovascular or the psychiatric condition which appeared first in individuals with both illnesses (Almhdawi et al., 2021). In addition, research in this area has mainly focused on the relationship between cardiovascular health and depression or generalized anxiety disorder, with little attention paid to other psychiatric conditions such as psychosis and bipolar disorder. Lastly, no studies have so far adequately accounted for family-related mechanisms that may be driving any observed associations, such as certain genetic backgrounds or early childhood environments. Now, in eLife, Unnur Valdimarsdóttir, Qing Shen and colleagues report the results of a study designed to address some of these limitations (Shen et al., 2022).

The team (who are based in China, the United States, Iceland and Sweden) used the Swedish Patient Register to identify nearly 0.9 million individuals recently diagnosed with cardiovascular disease, and with no prior history of psychiatric disorders. Throughout the study period, these patients were then followed until they first received a mental health diagnosis within the study period. In addition, the study included a remarkable family-comparison design, whereby participants’ siblings who had no mental health or cardiovascular conditions at the time of the diagnosis were also tracked over time. The risk of developing any psychiatric condition in both patients and siblings could therefore be compared. This approach allowed Shen et al. to control for familial factors that are often difficult to measure and, if left unaccounted for in study design, could contribute to a spurious association between cardiovascular disorders and subsequent mental illness.

The results indicate that, compared to their unaffected siblings, study participants were 2.7 times more at risk of developing a psychiatric disorder within a year of having received their diagnoses of cardiovascular illness (even after accounting for familial factors, prior history of psychiatric illness and sociodemographic variables such as age, sex or socioeconomic status). Similar associations were observed when study participants were compared to non-sibling controls. In addition, individuals who developed a psychiatric disorder during that first year had a 55% increased risk of dying from a heart-related condition compared to patients who retained good mental health. In this cohort, the co-occurrence of any mental illness therefore negatively impacted the course of cardiovascular diseases.

Despite its strengths, this work also has some limitations. Notably, smoking behaviour and alcohol consumption were not adequately controlled for, despite being directly and independently associated with cardiovascular disease and mental illnesses (Dani and Harris, 2005; Mukamal, 2006). Not accounting for either of these lifestyle factors could overestimate the true relationship between these two conditions. In addition, various psychiatric subtypes with distinct phenotypes were combined — for example, all types of anxiety conditions, from generalized anxiety to post-traumatic stress disorder, were merged into a single mental health outcome. Each of these disorders is likely to have specific associations with cardiovascular health, which could not be captured by this experimental design.

The work by Shen et al. highlights how important it is to monitor psychiatric symptoms while treating cardiovascular diseases. Their findings should encourage the scientific community to fill existing knowledge gaps. In particular, it is becoming increasingly clear that evidence derived from high-income countries, where most research is conducted, cannot be directly translated to other settings. For instance, age-standardized mortality rates for cardiovascular disease are mostly decreasing in European and North American populations, while suicide mortality (as an indicator of mental health burdens) rises with age. By contrast, cardiac mortality rates are rising in certain low- and middle-income countries such as Mexico and India, with suicide mortality occurring at younger ages (Reynales-Shigematsu et al., 2018; Ke et al., 2018; World Health Organization, 2022; Phillips and Cheng, 2012). Context-specific data will therefore need to be collected for cardiovascular diseases to be appropriately managed across the world through integrated healthcare approaches.
